# Torsional Low-Strain Test for Nondestructive Integrity Examination of Existing High-Pile Foundation

**DOI:** 10.3390/s22145330

**Published:** 2022-07-16

**Authors:** Yunpeng Zhang, M. Hesham El Naggar, Wenbing Wu, Zongqin Wang

**Affiliations:** 1 Faculty of Engineering, China University of Geosciences, Wuhan 430074, China; zypsky@cug.edu.cn (Y.Z.); wzongqin@cug.edu.cn (Z.W.); 2 Geotechnical Research Centre, Department of Civil and Environmental Engineering, Western University, London, ON N6A 5B9, Canada; helnaggar@eng.uwo.ca

**Keywords:** nondestructive test, existing pile integrity, low-strain test, wave propagation

## Abstract

Low-strain tests are widely utilized as a nondestructive approach to assess the integrity of newly piled foundations. So far, the examination of existing pile foundations is becoming an indispensable protocol for pile recycling or post-disaster safety assessment. However, the present low-strain test is not capable of testing existing pile foundations. In this paper, the torsional low-strain test (TLST) is proposed to overcome this drawback. Both the upward and downward waves are considered in the TLST wave propagation model established in this paper so that a firm theoretical basis is grounded for the test signal interpretations. A concise semi-analytical solution is derived and its rationality is verified by comparisons with the existing solutions for newly piled foundations and the finite element results. The main conclusions of this study can be drawn as follows: (1). by placing the sensors where the incident wave is applied, the number of reflected signals can be minimized; (2). the defects can be more evidently identified if the incident wave/sensors are input/installed close to the superstructure/pile head.

## 1. Introduction

Among many structure health monitoring approaches (static load test [1], image-based displacement measurement [2], low-strain test [3], and high-strain test [4]), the low-strain test is so far the most intuitive and economical way to assess the integrity of deep foundations, especially pile foundations [5,6,7,8]. This is because the test signal of the low-strain test is easily identifiable and it involves no disposable equipment or gauges. The traditional low-strain test utilizes longitudinal harmonic excitation as the incident wave so that an exposed cross-section of the foundation is needed to conduct the test [9,10]. Hence, the low-strain test is commonly used as the integrity inspection for newly piled foundations instead of existing ones. However, after decades of vigorous developments in infrastructure construction, the testing demands in major global construction markets have shifted from the newly piled foundations to the existing ones [11,12,13,14]. As a result, upgrading the low-strain test to satisfy the testing of existing foundations is especially urgent.

The fundamental theory of the low-strain test for pile foundations originates from the longitudinal vibration theory of the pile [15,16]. The combination of one-dimensional rod theory and the subgrade reaction model forms the mathematical prototype of the low-strain test [17,18,19]. High-frequency interferences often occur during the tests of large diameter piles, which are not revealed by the one-dimensional rod theory. The high-frequency interference can be addressed by simulating the soil and pile as three-dimensional continuum media [20,21]. However, due to the massive computation involved in rigorous 3D continuum models, digital signal filters (e.g., Savitzky–Golay) are preferred by engineers. Compared to the longitudinal vibration of piles, torsional vibrations receive less attention because they are not that common in nature. For most studies, torsional vibrations of piles are only regarded as additional problems caused by eccentric loadings [22]. However, since torsional vibration is less common in nature than longitudinal or horizontal vibration, it is an ideal subject for studying pile testing, as its strain wave signal may not be easily jammed or suppressed by other environmental loads. Moreover, because the velocity of the torsional wave is much smaller than that of the longitudinal wave, the torsional low-strain test has a smaller detection blind zone than the traditional low-strain test [23,24]. The torsional vibration theory is initially established on a similar basis to the longitudinal one: by simplifying the soil medium to infinitely thin layers, the rigorous 3D continuum theory for soil medium can be reduced to the plane strain model, based on which the straightforward closed-form solutions can be derived [25,26,27,28,29]. As the torsional vibration of pile foundations gained interest in the most recent decade, the finite element method (FEM) [30,31,32], finite integration technique [33], and boundary element method [34,35] all considerably fulfilled the knowledge of wave propagation across the soil-pile system during vibration.

In the literature mentioned above, the torsional incident wave is input at the pile head, under which circumstance there will only be an upward wave or a downward wave inside the intact pile body at the same time and neither will exist simultaneously [36,37,38]. However, when conducting the test for existing high-pile foundations, the incident wave can only be input at the shaft of the pile, because the pile head is fixed into the superstructure firmly. As a result, the upward and downward waves propagate inside the pile body simultaneously, dramatically increasing the complexity of strain wave signals. To account for this phenomenon, a rigorous torsional wave propagation model, taking both the upward and downward waves into account, is established in this paper to guide the signal interpretations of the TLST for existing high-pile foundations. Based on the proposed model, the optimal excitation and signal receiving layouts in the TLST for existing high-pile foundations are revealed.

## 2. Mathematical Model and Assumptions

**Figure 1:**
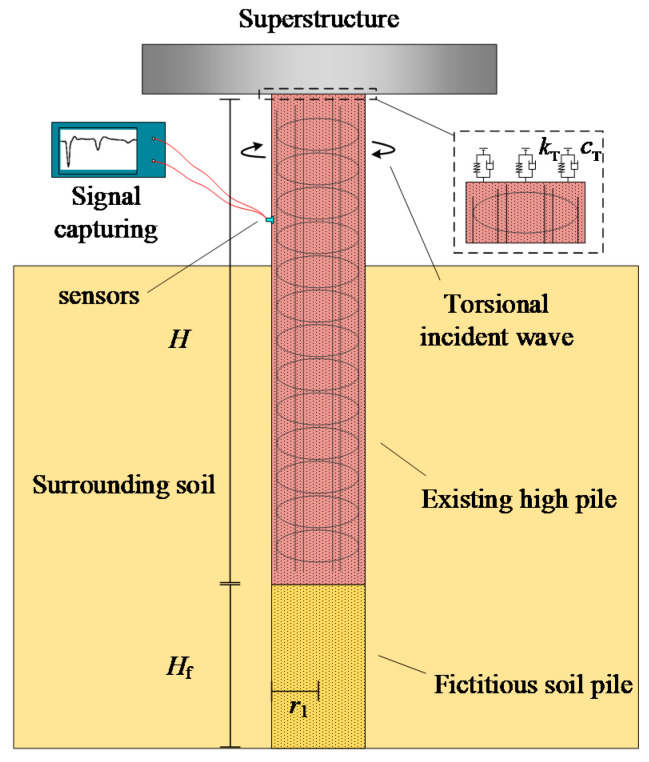
Schematics of torsional low-strain test for existing high-pile foundations.

The layout of the TLST for the existing high-pile foundation is depicted in Figure 1. Due to the head of the existing pile being firmly fixed in the superstructure, the torsional incident wave can only be input at the extending pile shaft so that both the upward and downward strain waves are generated. The pile is modeled as a one-dimensional rod in the proposed mathematical model and the surrounding soil is modeled as a three-dimensional viscoelastic medium. The interactions between the pile and the superstructure are simplified to springs and dashpots. Further, the fictitious soil-pile model [28] is introduced herein to authentically simulate the wave reflection at the interface of the pile bottom and the pile end soil. Other general assumptions adopted are listed as follows:

1. Throughout the TLSTs, the soil-pile system only undergoes small strain deformations so that the surrounding soil and the pile shaft are assumed to remain in perfect contact.
2. The incident wave utilized as the input of TLSTs in this paper is a half-sine harmonic impulse.
3. There are no normal and shear stresses at the ground surface and the amplitude of the strain wave diminishes to zero in radial infinity in the soil.
4. The displacement and forces at the interfaces of the fictitious soil pile and the real pile are continuous. By increasing the length and modulus of the fictitious soil pile, the foundation can shaft from the end-bearing piles to the floating piles.
5. The velocity response at the pile shaft is acquired to simulate the test results collected from the velocity or acceleration sensors installed at the pile shaft.

## 3. Governing Equations and Boundary Conditions

### 3.1. Governing Equations

Based on the continuum theories, the equilibrium equations for the soil medium in a cylindrical coordinate system can be written as

(1) $$\left(G_{j}^{s}+\eta_{j}^{s}\frac{\partial }{\partial t}\right)\nabla^{2}u_{j}^{s}(z,r,t)=\rho_{j}^{s}\frac{\partial^{2}u_{j}^{s}(z,r,t)}{\partial t^{2}}$$

where $G_{j}^{s}$, $\eta_{j}^{s}$, $u_{j}^{s}$, and $\rho_{j}^{s}$ denote the shear modulus, material damping, circumferential displacement, and density of the *j*th (vertically labeled) soil layer, respectively. $\nabla^{2}=\frac{\partial^{2}}{\partial r^{2}}+\frac{\partial }{r\partial r}+\frac{\partial^{2}}{\partial z^{2}}-\frac{1}{r^{2}}$ is the Laplacian written in the cylindrical coordinates.

The three-dimensional rod theory can better reveal the wave propagation during the TLSITs. However, its adoption would significantly increase the mathematical complexity of the problem, resulting in terrible computational efficiency. Further, it was reported by Zhang et al. [24] that the wave signal captured at the pile edge is limitedly influenced by the three-dimensional effect during the TLSITs. Hence, the pile is modeled through the one-dimensional rod theory in pursuit of a more efficient closed-form solution. Commonly, the high-pile foundation can be divided into two parts: one embedded in the soil, the other one extending out of the soil. For the part that is embedded in the soil, the equilibrium equation can be written as

(2) $$\left(G_{j}^{p}I_{j}^{p}+\eta_{j}^{p}I_{j}^{p}\frac{\partial }{\partial t}\right)\frac{\partial^{2}\varphi_{j}^{p}(z,t)}{\partial z^{2}}-2\pi r_{1}^{2}f_{j}^{s}(z,t)=\rho_{j}^{p}I_{j}^{p}\frac{\partial^{2}\varphi_{j}^{p}(z,t)}{\partial t^{2}}$$

For the part extending out of the soil, the equilibrium equation can be written as

(3) $$\left(G_{m}^{p}I_{m}^{p}+\eta_{m}^{p}I_{m}^{p}\frac{\partial }{\partial t}\right)\frac{\partial^{2}\varphi_{m}^{p}(z,t)}{\partial z^{2}}=\rho_{m}^{p}I_{m}^{p}\frac{\partial^{2}\varphi_{m}^{p}(z,t)}{\partial t^{2}}$$

where $G_{j}^{p}$, $\eta_{j}^{p}$, $I_{j}^{p}$, $\varphi_{j}^{p}$, $\rho_{j}^{p}$, $f_{j}^{s}$, and $r_{1}^{}$ are the shear modulus, material damping, polar moment of inertia, twist angle, density, pile-side resistance, and the radius of the *j*th pile segment.

### 3.2. Boundary and Initial Conditions

The displacement and stress in the soil medium diminish at the radial infinite so that the following boundary conditions can be acquired:

(4) $${u_{j}^{s}(z,r,t)|}_{r\to \infty }=0$$

(5) $${\tau_{j}^{s}(z,r,t)|}_{r\to \infty }=0$$

The interactions between the soil layers are simulated by a distributed Kelvin–Voigt model, whose formulas can be presented as

(6) $${\left[\left(G_{j}^{s}+\eta_{j}^{s}\frac{\partial }{\partial t}\right)\frac{\partial u_{j}^{s}(z,r,t)}{\partial z}-\left(k_{j}+c_{j}\frac{\partial }{\partial t}\right)u_{j}^{s}(z,r,t)\right]|}_{z=h_{j}}=0$$

(7) $${\left[\left(G_{j}^{s}+\eta_{j}^{s}\frac{\partial }{\partial t}\right)\frac{\partial u_{j}^{s}(z,r,t)}{\partial z}+\left(k_{j-1}+c_{j-1}\frac{\partial }{\partial t}\right)u_{j}^{s}(z,r,t)\right]|}_{z=h_{j-1}}=0$$

The transient impulse is subjected to the side of the extending part. Considering that the stress distribution inside the pile shaft is continuous, these boundary conditions can be written as

(8) $${\left[\left(G_{m}^{p}I_{m}^{p}+\eta_{m}^{p}I_{m}^{p}\frac{\partial }{\partial t}\right)\frac{\partial \varphi_{m}^{p}(z,t)}{\partial z}\right]|}_{z=h_{m}}+T(t)={\left[\left(G_{m+1}^{p}I_{m+1}^{p}+\eta_{m+1}^{p}I_{m+1}^{p}\frac{\partial }{\partial t}\right)\frac{\partial \varphi_{m}^{p}(z,t)}{\partial z}\right]|}_{z=h_{m}}$$

(9) $${\varphi_{j}^{p}|}_{z=h_{j}}={\varphi_{j+1}^{p}|}_{z=h_{j}}$$

(10) $${\tau_{j}^{p}|}_{z=h_{j}}={\tau_{j+1}^{p}|}_{z=h_{j}}$$

The pile end soil-pile end interaction is modeled by the fictitious soil pile. At the end of the fictitious soil pile, the displacement is supposed to be zero.

(11) $${\varphi_{1}^{\mathrm{fp}}(z,t)|}_{z=L}=0$$

The interaction between the pile and the upper structure is simplified to elastic springs and dashpots:

(12) $${\left[\left(G_{m+1}^{p}I_{m+1}^{p}+\eta_{m+1}^{p}I_{m+1}^{p}\frac{\partial }{\partial t}\right)\frac{\partial \varphi_{m}^{p}(z,t)}{\partial z}+\left(k_{T}+c_{T}\frac{\partial }{\partial t}\right)\varphi_{m}^{p}(z,t)\right]|}_{z=0}=0$$

where $k_{T}$ and $c_{T}$ denote the elastic and damping coefficients of the springs and dashpots, respectively.

When conducting the low-strain integrity test, both the soil and pile only go through tiny deformations, under which circumstance the motions of the soil and pile can be regarded as simultaneous.

(13) $${\varphi_{j}^{p}(z,t)|}_{r=r_{1}}\cdot r_{1}={u_{j}^{s}(z,r,t)|}_{r=r_{1}}$$

At the initial moment, the system has no velocity nor acceleration, and the transient pile-side impulse is the only reason for the system vibration.

(14) $${u_{j}^{s}(z,r,t)|}_{t=0}=0$$

(15) $${\frac{\partial u_{j}^{s}(z,r,t)}{\partial t}|}_{t=0}=0$$

(16) $${\varphi_{j}^{p}(z,t)|}_{t=0}=0$$

(17) $${\frac{\partial \varphi_{j}^{p}(z,t)}{\partial t}|}_{t=0}=0$$

## 4. Solution of Dynamic Equilibrium Equations

### 4.1. Solution of the Governing Equation in the Surrounding Soil

By performing Laplace Transform on both sides of Equation (1) and conducting the variable separation method, Equation (1) can degenerate to the following two differential equations:

(18) $$r^{2}R_{j}^{s}{}^{\prime \prime }(r,s)+rR_{j}^{s}{}^{\prime }(r,s)-\left[\kappa_{j}^{2}r^{2}+1\right]R_{j}^{s}(r,s)=0$$

(19) $$Z_{j}^{s}{}^{\prime \prime }(z,s)+\beta_{j}^{2}Z_{j}^{s}(z,s)=0$$

where $\kappa_{j}^{2}=\frac{\rho_{j}^{s}s^{2}}{G_{j}^{s}+\eta_{j}^{s}s}+\beta_{j}^{2}$. Therefore, the general solution of Equation (1) can be acquired through the combination of general solutions of Equations (18) and (19) as

(20) $$U_{j}^{s}(z,r,s)=\left[E_{j}^{}K_{1}\left(\kappa_{j}^{}r\right)+F_{j}^{}I_{1}\left(\kappa_{j}^{}r\right)\right]\cdot \left[M_{j}^{}\sin(\beta_{j}^{}z)+N_{j}^{}\cos(\beta_{j}^{}z)\right]$$

where $U_{j}^{s}$ is the Laplace Transform of $u_{j}^{s}$, while $E_{j}^{}$, $F_{j}^{}$, $M_{j}^{}$ and $N_{j}^{}$ are all undetermined coefficients. Meanwhile, $I_{1}\left(\cdot \right)$ and $K_{1}\left(\cdot \right)$ are modified Bessel Function of order one of the first and second kind, respectively.

Submitting Equation (20) into Equations (4) and (5), one obtains

(21) $$U_{j}^{s}(z,r,s)=\left[M_{j}^{}\sin(\beta_{j}^{}z)+N_{j}^{}\cos(\beta_{j}^{}z)\right]\cdot K_{1}\left(\kappa_{j}^{}r\right)$$

Further considering the interaction between soil layers, as listed in Equations (6) and (7), the following transcendental equations can be established:

(22) $$G_{j}^{s*}\beta_{j}^{2}\left[\tan(\beta_{j}^{}h_{j})-\tan(\beta_{j}^{}h_{j-1})\right]+G_{j}^{s**}\beta_{j}^{}\left[\tan(\beta_{j}^{}h_{j})\tan(\beta_{j}^{}h_{j-1})+1\right]+\left[\tan(\beta_{j}^{}h_{j-1})+\tan(\beta_{j}^{}h_{j})\right]=0$$

where $G_{j}^{s*}=\frac{{\left(G_{j}^{s}+\eta_{j}^{s}s\right)}^{2}}{\left(k_{j}+c_{j}s\right)\left(k_{j-1}+c_{j-1}s\right)}$ and $G_{j}^{s**}=\frac{\left(G_{j}^{s}+\eta_{j}^{s}s\right)\left(k_{j}+c_{j}s+k_{j-1}+c_{j-1}s\right)}{\left(k_{j}+c_{j}s\right)\left(k_{j-1}+c_{j-1}s\right)}$. With the introduction of local coordinates $\left[0,l_{j}\right]$, the transcendental equation can be simplified to

(23) $$\tan(\beta_{j}^{}l_{j})-\frac{\left(G_{j}^{s}+\eta_{j}^{s}s\right)\left(k_{j}+c_{j}s+k_{j-1}+c_{j-1}s\right)\beta_{j}^{}}{{\left(G_{j}^{s}+\eta_{j}^{s}s\right)}^{2}\beta_{j}^{2}-\left(k_{j}+c_{j}s\right)\left(k_{j-1}+c_{j-1}s\right)}=0$$

Through numerical iterations, the above transcendental equations can be solved with a series of numerical answers, which can be denoted as $\beta_{j1}^{}$, $\beta_{j2}^{}$, $\beta_{j3}^{}$, …,$\beta_{jn}^{}$. Then, Equation (21) can be written as

(24) $$U_{j}^{s}(z,r,s)=\sum_{n=1}^{\infty }A_{jn}^{}\sin(\beta_{jn}^{}z+\varphi_{jn}^{})\cdot K_{1}\left(\kappa_{jn}^{}r\right)$$

where $A_{jn}^{}=\sqrt{M_{jn}^{2}+N_{jn}^{2}}$, $\varphi_{jn}^{}=\arctan\left(\frac{N_{jn}^{}}{M_{jn}^{}}\right)$, $\frac{M_{jn}^{s}}{N_{jn}^{s}}=\frac{\left(k_{j}+c_{j}s\right)}{\left(G_{j}^{s}+\eta_{j}^{s}s\right)\beta_{jn}^{}}$. The resistance force of soil acting on the pile side can be expressed as

(25) $$f_{j}^{s}=\left(G_{j}^{s}+\eta_{j}^{s}s\right)\sum_{n=1}^{\infty }A_{jn}^{}\kappa_{jn}^{}\sin(\beta_{jn}^{}z+\varphi_{jn}^{})K_{2}\left(\kappa_{jn}^{}r_{1}\right)$$

### 4.2. Solution of the Governing Equation of the Pile

Similarly, by performing Laplace Transform on both sides of Equations (2) and (3), one obtains

(26) $$\left(G_{j}^{p}I_{j}^{p}+\eta_{j}^{p}I_{j}^{p}s\right)\frac{\partial^{2}\phi_{j}^{p}(z,s)}{\partial z^{2}}-2\pi r_{1}^{2}f_{j}^{s}(z,t)=\rho_{j}^{p}I_{j}^{p}s^{2}\phi_{j}^{p}(z,s)$$

(27) $$\left(G_{m}^{p}I_{m}^{p}+\eta_{m}^{p}I_{m}^{p}s\right)\frac{\partial^{2}\phi_{m}^{p}(z,s)}{\partial z^{2}}=\rho_{m}^{p}I_{m}^{p}s^{2}\phi_{m}^{p}(z,s)$$

where $\phi_{j}^{p}$ is the Laplace Transform of $\varphi_{j}^{p}$ with respect to *t*. It can be found that Equations (26) and (27) are non-homogeneous and homogeneous functions, respectively. The general solution of the corresponding homogeneous function of Equation (26) can be given as

(28) $$\phi_{j}^{p}(z,s)=C_{j}^{p}\sin(\lambda_{j}z)+D_{j}^{p}\cos(\lambda_{j}z)$$

where $\lambda_{j}=\sqrt{-\frac{\rho_{j}^{p}s^{2}}{G_{j}^{p}+\eta_{j}^{p}s}}$. The specific solution of Equation (26) is found as

(29) $$\sum_{n=1}^{\infty }A_{jn}^{}\kappa_{jn}^{}k_{jn}^{s}\sin(\beta_{jn}^{}z+\varphi_{jn}^{})\cdot K_{2}\left(\kappa_{jn}^{}r_{1}\right)$$

where $k_{jn}^{s}=-\frac{2\pi r_{1}^{2}\left(G_{j}^{s}+\eta_{j}^{s}s\right)}{\left(G_{j}^{p}I_{j}^{p}+\eta_{j}^{p}I_{j}^{p}s\right)\beta_{jn}^{2}+\rho_{j}^{p}I_{j}^{p}s^{2}}$. The general solutions for the buried and extending pile segments can then be written as

Embedded pile segments:

(30) $$\begin{array}{l} \phi_{j}^{p}(z,s)=C_{j}^{p}\sin(\lambda_{j}z)+D_{j}^{p}\cos(\lambda_{j}z)\\ +\sum_{n=1}^{\infty }A_{jn}^{}\kappa_{jn}^{}k_{jn}^{s}\sin(\beta_{jn}^{}z+\varphi_{jn}^{})\cdot K_{2}\left(\kappa_{jn}^{}r_{1}\right) \end{array}$$

Extending pile segments:

(31) $$\phi_{m}^{p}(z,s)=C_{m}^{p}\sin(\lambda_{m}z)+D_{m}^{p}\cos(\lambda_{m}z)$$

Based on the small strain assumption, the displacements at the soil-pile interface for embedded pile segments are continuous. Next, Equation (30) is substituted into (13), herein introducing the orthogonality of the following equations:

(32) $$\int_{0}^{l_{j}}\sin(\beta_{jn}^{}z+\varphi_{jn}^{})\sin(\beta_{in}^{}z+\varphi_{in}^{})dz=\{\begin{array}{l} 0,\;j\neq i\\ \frac{l_{j}}{2}-\frac{\sin(2\beta_{jn}^{}l_{j}+2\varphi_{jn}^{})-\sin(2\varphi_{jn}^{})}{4\beta_{jn}^{}},j=i \end{array}$$

With the utilization of Equation (32), one obtains

(33) $$A_{jn}^{}\kappa_{jn}^{}=C_{j}^{p}\delta_{jn1}^{}+D_{j}^{p}\delta_{jn2}^{}$$

In addition, the undetermined coefficients $C_{j}^{p}$ and $D_{j}^{p}$, and other parameters, can be derived from the following relations:

(34) $$\delta_{jn1}^{}=\frac{r_{1}\kappa_{jn}^{}\chi_{jn1}^{}}{\chi_{jn3}^{}\left[K_{1}\left(\kappa_{jn}^{}r\right)-k_{jn}^{s}r_{1}K_{2}\left(\kappa_{jn}^{}r_{1}\right)\right]}$$

(35) $$\delta_{jn2}^{}=\frac{r_{1}\kappa_{jn}^{}\chi_{ji2}^{}}{\chi_{jn3}^{}\left[K_{1}\left(\kappa_{jn}^{}r\right)-k_{jn}^{s}r_{1}K_{2}\left(\kappa_{jn}^{}r_{1}\right)\right]}$$

(36) $$\chi_{jn1}^{}=\frac{1}{2}\cdot \left[\frac{\sin(\lambda_{j}l_{j}-\beta_{jn}^{}l_{j}-\varphi_{jn}^{})+\sin(\varphi_{jn}^{})}{\left(\lambda_{j}-\beta_{jn}^{}\right)}-\frac{\sin(\lambda_{j}l_{j}+\beta_{jn}^{}l_{j}+\varphi_{jn}^{})-\sin(\varphi_{jn}^{})}{\left(\lambda_{j}+\beta_{jn}^{}\right)}\right]$$

(37) $$\chi_{ji2}^{}=\frac{1}{2}\cdot \left[\frac{\cos(\lambda_{j}l_{j}-\beta_{jn}^{}l_{j}-\varphi_{jn}^{})-\cos(\varphi_{jn}^{})}{\left(\lambda_{j}-\beta_{jn}^{}\right)}-\frac{\cos(\lambda_{j}l_{j}+\beta_{jn}^{}l_{j}+\varphi_{jn}^{})-\cos(\varphi_{jn}^{})}{\left(\lambda_{j}+\beta_{jn}^{}\right)}\right]$$

(38) $$\chi_{jn3}^{}=\frac{l_{j}}{2}-\frac{\sin(2\beta_{jn}^{}l_{j}+2\varphi_{jn}^{})-\sin(2\varphi_{jn}^{})}{4\beta_{jn}^{}}$$

After the above derivation, the general solution for the embedded pile segments can then be given in a homogeneous equation form.

(39) $$\begin{array}{l} \phi_{j}^{p}(z,s)=C_{j}^{p}\left[\sin(\lambda_{j}z)+\sum_{n=1}^{\infty }k_{jn}^{s}\delta_{jn1}^{}\sin(\beta_{jn}^{}z+\varphi_{jn}^{})\cdot K_{2}\left(\kappa_{jn}^{}r_{1}\right)\right]\\ +D_{j}^{p}\left[\cos(\lambda_{j}z)+\sum_{n=1}^{\infty }k_{jn}^{s}\delta_{jn2}^{}\sin(\beta_{jn}^{}z+\varphi_{jn}^{})\cdot K_{2}\left(\kappa_{jn}^{}r_{1}\right)\right] \end{array}$$

To acquire the undetermined coefficients ($C_{j}^{p}$ and $D_{j}^{p}$), the continuous deformation boundary conditions at pile segment interfaces are utilized. Substituting Equations (30) and (31) into Equations (9) and (10), the iteration relations between different embedded pile segments can be obtained as

(40) $$\left[\begin{array}{c} C_{j+1}^{p}\\ D_{j+1}^{p} \end{array}\right]={\left[\begin{array}{cc} \psi_{j+1,1}(l_{j+1}) & \psi_{j+1,2}(l_{j+1})\\ \psi_{j+1,3}(l_{j+1}) & \psi_{j+1,4}(l_{j+1}) \end{array}\right]}^{-1}\times \left[\begin{array}{cc} \psi_{j1}(0) & \psi_{j2}(0)\\ \psi_{j3}(0) & \psi_{j4}(0) \end{array}\right]\left[\begin{array}{c} C_{j}^{p}\\ D_{j}^{p} \end{array}\right]$$

(41) $$\psi_{j1}(z)=\sin(\lambda_{j}z)+\sum_{n=1}^{\infty }\delta_{jn1}^{}\zeta_{jn}^{1}\left(z\right)$$

(42) $$\psi_{j2}(z)=\cos(\lambda_{j}z)+\sum_{n=1}^{\infty }\delta_{jn2}^{}\zeta_{jn}^{1}\left(z\right)$$

(43) $$\psi_{j3}(z)=G_{j}^{p*}\left[\lambda_{j}\cos(\lambda_{j}z)+\sum_{n=1}^{\infty }\delta_{jn1}^{}\zeta_{jn}^{2}\left(z\right)\right]$$

(44) $$\psi_{j4}(z)=G_{j}^{p*}\left[-\lambda_{j}\sin(\lambda_{j}z)+\sum_{n=1}^{\infty }\delta_{jn2}^{}\zeta_{jn}^{2}\left(z\right)\right]$$

(45) $$\zeta_{jn}^{1}\left(z\right)=k_{jn}^{s}\sin(\beta_{jn}^{}z+\varphi_{jn}^{})\cdot K_{2}\left(\kappa_{jn}^{}r_{1}\right)$$

(46) $$\zeta_{jn}^{2}\left(z\right)=k_{jn}^{s}\beta_{jn}^{}\cos(\beta_{jn}^{}z+\varphi_{jn}^{})\cdot K_{2}\left(\kappa_{jn}^{}r_{1}\right)$$

(47) $$G_{j}^{p*}=G_{j}^{p}I_{j}^{p}+\eta_{j}^{p}I_{j}^{p}s$$

Similarly, the coefficient transform relations between the embedded and the extending pile segments can be expressed as

(48) $$\left[\begin{array}{c} C_{m}^{p}\\ D_{m}^{p} \end{array}\right]={\left[\begin{array}{cc} \sin(\lambda_{m}l_{m}) & \cos(\lambda_{m}l_{m})\\ G_{m}^{p*}\lambda_{m}\cos(\lambda_{m}l_{m}) & -G_{m}^{p*}\lambda_{m}\sin(\lambda_{m}l_{m}) \end{array}\right]}^{-1}\times \left[\begin{array}{cc} \psi_{m-1,1}(0) & \psi_{m-1,2}(0)\\ \psi_{m-1,3}(0) & \psi_{m-1,4}(0) \end{array}\right]\left[\begin{array}{c} C_{m-1}^{p}\\ D_{m-1}^{p} \end{array}\right]$$

The continuous stress conditions at the location of the pile side impulse can be written as

(49) $$\begin{array}{l} \left[\begin{array}{c} C_{m+1}^{p}\\ D_{m+1}^{p} \end{array}\right]={\left[\begin{array}{cc} \sin(\lambda_{m+1}l_{m+1}) & \cos(\lambda_{m+1}l_{m+1})\\ G_{m+1}^{p*}\lambda_{m+1}\cos(\lambda_{m+1}l_{m+1}) & -G_{m+1}^{p*}\lambda_{m+1}\sin(\lambda_{m+1}l_{m+1}) \end{array}\right]}^{-1}\times \left[\begin{array}{cc} 0 & 1\\ G_{m}^{p*}\lambda_{m} & 0 \end{array}\right]\left[\begin{array}{c} C_{m}^{p}\\ D_{m}^{p} \end{array}\right]\\ +{\left[\begin{array}{cc} \sin(\lambda_{m+1}l_{m+1}) & \cos(\lambda_{m+1}l_{m+1})\\ G_{m+1}^{p*}\lambda_{m+1}\cos(\lambda_{m+1}l_{m+1}) & -G_{m+1}^{p*}\lambda_{m+1}\sin(\lambda_{m+1}l_{m+1}) \end{array}\right]}^{-1}\left[\begin{array}{c} 0\\ T(\omega ) \end{array}\right] \end{array}$$

where $T(\omega )=\frac{T}{\pi^{2}-T^{2}\omega^{2}}(1+e^{-i\omega T})$ represents the half-sine harmonic impulse acted on the pile shaft in the frequency domain. Combing the boundary conditions at the pile top and end, one obtains

(50) $$\frac{C_{1}^{p}}{D_{1}^{p}}=-\frac{\psi_{1,2}(l_{j}^{})}{\psi_{1,1}(l_{j}^{})}$$

(51) $$\frac{C_{m+1}^{p}}{D_{m+1}^{p}}=-\frac{k_{T}+c_{T}s}{G_{m+1}^{p*}\lambda_{m+1}}$$

The deformation and stress at the interfaces of different pile segments are continuous so that

(52) $$\left[\begin{array}{c} C_{m+1}^{p}\\ D_{m+1}^{p} \end{array}\right]=\left[\begin{array}{cc} \chi_{1} & \chi_{2}\\ \chi_{3} & \chi_{4} \end{array}\right]\left[\begin{array}{c} C_{1}^{p}\\ D_{1}^{p} \end{array}\right]+\left[\begin{array}{c} \mu_{1}\\ \mu_{2} \end{array}\right]$$

where the matrices $\left[\begin{array}{cc} \chi_{1} & \chi_{2}\\ \chi_{3} & \chi_{4} \end{array}\right]$ and $\left[\begin{array}{c} \mu_{1}\\ \mu_{2} \end{array}\right]$ can be derived from

(53) $$\begin{array}{l} \left[\begin{array}{cc} \chi_{1} & \chi_{2}\\ \chi_{3} & \chi_{4} \end{array}\right]={\left[\begin{array}{cc} \sin(\lambda_{m+1}l_{m+1}) & \cos(\lambda_{m+1}l_{m+1})\\ G_{m+1}^{p*}\lambda_{m+1}\cos(\lambda_{m+1}l_{m+1}) & -G_{m+1}^{p*}\lambda_{m+1}\sin(\lambda_{m+1}l_{m+1}) \end{array}\right]}^{-1}\\ \times \left[\begin{array}{cc} 0 & 1\\ G_{m}^{p*}\lambda_{m} & 0 \end{array}\right]{\left[\begin{array}{cc} \sin(\lambda_{m}l_{m}) & \cos(\lambda_{m}l_{m})\\ G_{m}^{p*}\lambda_{m}\cos(\lambda_{m}l_{m}) & -G_{m}^{p*}\lambda_{m}\sin(\lambda_{m}l_{m}) \end{array}\right]}^{-1}\\ \times \left[\begin{array}{cc} \psi_{m-1,1}(0) & \psi_{m-1,2}(0)\\ \psi_{m-1,3}(0) & \psi_{m-1,4}(0) \end{array}\right]\ldots \ldots {\left[\begin{array}{cc} \psi_{3,1}(l_{j+1}) & \psi_{3,2}(l_{j+1})\\ \psi_{3,3}(l_{j+1}) & \psi_{3,4}(l_{j+1}) \end{array}\right]}^{-1}\\ \times \left[\begin{array}{cc} \psi_{2,1}(0) & \psi_{2,2}(0)\\ \psi_{2,3}(0) & \psi_{2,4}(0) \end{array}\right]{\left[\begin{array}{cc} \psi_{2,1}(l_{j+1}) & \psi_{2,2}(l_{j+1})\\ \psi_{2,3}(l_{j+1}) & \psi_{2,4}(l_{j+1}) \end{array}\right]}^{-1}\times \left[\begin{array}{cc} \psi_{1,1}(0) & \psi_{1,2}(0)\\ \psi_{1,3}(0) & \psi_{1,4}(0) \end{array}\right] \end{array}$$

(54) $$\left[\begin{array}{c} \mu_{1}\\ \mu_{2} \end{array}\right]={\left[\begin{array}{cc} \sin(\lambda_{m+1}l_{m+1}) & \cos(\lambda_{m+1}l_{m+1})\\ G_{m+1}^{p*}\lambda_{m+1}\cos(\lambda_{m+1}l_{m+1}) & -G_{m+1}^{p*}\lambda_{m+1}\sin(\lambda_{m+1}l_{m+1}) \end{array}\right]}^{-1}\left[\begin{array}{c} 0\\ T(\omega ) \end{array}\right]$$

Equation (52) can be further simplified to

(55) $$C_{m+1}^{p}=\chi_{1}C_{1}^{p}+\chi_{2}D_{1}^{p}+\mu_{1}$$

(56) $$D_{m+1}^{p}=\chi_{3}C_{1}^{p}+\chi_{4}D_{1}^{p}+\mu_{2}$$

in which,

(57) $$C_{1}^{p}=\frac{G_{m+1}^{p*}\lambda_{m+1}\mu_{1}+\left(k_{T}+c_{T}s\right)\mu_{2}}{\left[G_{m+1}^{p*}\lambda_{m+1}\chi_{2}+\left(k_{T}+c_{T}s\right)\chi_{4}\right]\frac{\psi_{1,1}(l_{j}^{})}{\psi_{1,2}(l_{j}^{})}-\left[G_{m+1}^{p*}\lambda_{m+1}\chi_{1}+\left(k_{T}+c_{T}s\right)\chi_{3}\right]}$$

(58) $$D_{1}^{p}=\frac{G_{m+1}^{p*}\lambda_{m+1}\mu_{1}+\left(k_{T}+c_{T}s\right)\mu_{2}}{\left[G_{m+1}^{p*}\lambda_{m+1}\chi_{1}+\left(k_{T}+c_{T}s\right)\chi_{3}\right]\frac{\psi_{1,2}(l_{j}^{})}{\psi_{1,1}(l_{j}^{})}-\left[G_{m+1}^{p*}\lambda_{m+1}\chi_{2}+\left(k_{T}+c_{T}s\right)\chi_{4}\right]}$$

(59) $$C_{m+1}^{p}=\left[\chi_{2}-\chi_{1}\frac{\psi_{1,2}(l_{j}^{})}{\psi_{1,1}(l_{j}^{})}\right]D_{1}^{p}+\mu_{1}$$

(60) $$D_{m+1}^{p}=\left[\chi_{4}-\chi_{3}\frac{\psi_{1,2}(l_{j}^{})}{\psi_{1,1}(l_{j}^{})}\right]D_{1}^{p}+\mu_{2}$$

Then, the undetermined coefficients of the near-ground pile segment can be acquired through the inverse transfer function as

(61) $$\begin{array}{l} \left[\begin{array}{c} C_{m}^{p}\\ D_{m}^{p} \end{array}\right]={\left[\begin{array}{cc} 0 & 1\\ G_{m}^{p*}\lambda_{m} & 0 \end{array}\right]}^{-1}\left[\begin{array}{cc} \sin(\lambda_{m+1}l_{m+1}) & \cos(\lambda_{m+1}l_{m+1})\\ G_{m+1}^{p*}\lambda_{m+1}\cos(\lambda_{m+1}l_{m+1}) & -G_{m+1}^{p*}\lambda_{m+1}\sin(\lambda_{m+1}l_{m+1}) \end{array}\right]\\ \times \left[\begin{array}{c} C_{m+1}^{p}\\ D_{m+1}^{p} \end{array}\right]-{\left[\begin{array}{cc} 0 & 1\\ G_{m}^{p*}\lambda_{m} & 0 \end{array}\right]}^{-1}\left[\begin{array}{c} 0\\ T(\omega ) \end{array}\right] \end{array}$$

The twist angle and velocity response of the near-ground pile segment can be obtained as

(62) $$\phi_{m}^{p}(z,s)=C_{m}^{p}\sin(\lambda_{m}z)+D_{m}^{p}\cos(\lambda_{m}z)$$

(63) $$V_{m}^{p}(z,t)=\frac{1}{2\pi }\int_{-\infty }^{+\infty }\phi_{m}^{p}(z,s)\cdot s\cdot e^{i\omega t}d\omega$$

## 5. Model Verification

To verify the correctness of the proposed model, the results calculated from the present solution are compared with those derived from the TLST theory aimed at the newly piled foundation and those computed from the finite element method (FEM). The soil-pile parameters utilized in this section are presented in Table 1 and Table 2.

### 5.1. Comparisons with the TLIST Signals of Newly Piled Foundations

**Figure 2:**
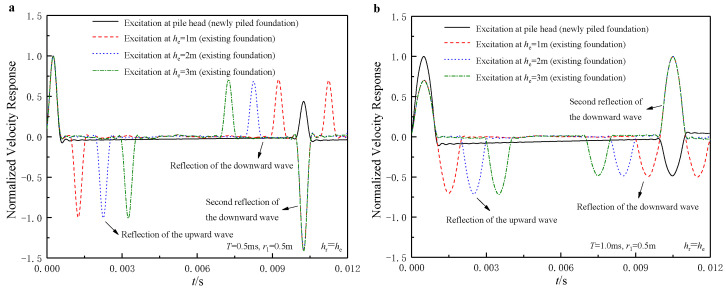
Comparisons of velocity response between existing and newly piled foundations: (**a**) viscoelastic boundary at the pile end (*T* = 0.5 ms); (**b**) fixed boundary at the pile end (*T* = 1.0 ms).

As mentioned, the classic TLST theory is established for newly piled foundations. Consequently, it is only capable of simulating the specific testing case in which the incident wave is input at the pile head. Unlike the newly piled foundations, the selections of incident wave input and signal receiving locations can be diverse for the testing of existing high-pile foundations. $h_{e}$ and $h_{r}$ are defined as the distances from the incident wave input location and the signal receiving location to the pile head. By adopting the soil and pile parameters in Table 1 and Table 2, the present solution is compared with the classic TLST theory established in Ref. [28]. The length of the fictitious soil pile is set as zero to simulate an end-bearing condition. As shown in Figure 2, the reflection of the upward wave at the pile head would result in an inverse wave signal after the incident wave. Further, as the incident wave is input more away from the pile head, the time intervals between the incident wave and the reflection of the upward wave would increase. Once the incident wave is input close enough to the pile head, the incident wave and the reflection of the upward wave will combine into one signal. It is also noticed that the reflection at the pile end in the newly piled foundation signal would always match the second reflection of the downward wave in the existing foundation signal, as long as the sensors are installed in the same place as the input of the incident wave. This is because the distance traveled by the strain wave at this time is exactly equal to twice the length of the pile, as the reflection at the pile end in the newly piled foundation signal does.

### 5.2. Comparisons with the FEM Results

**Figure 3:**
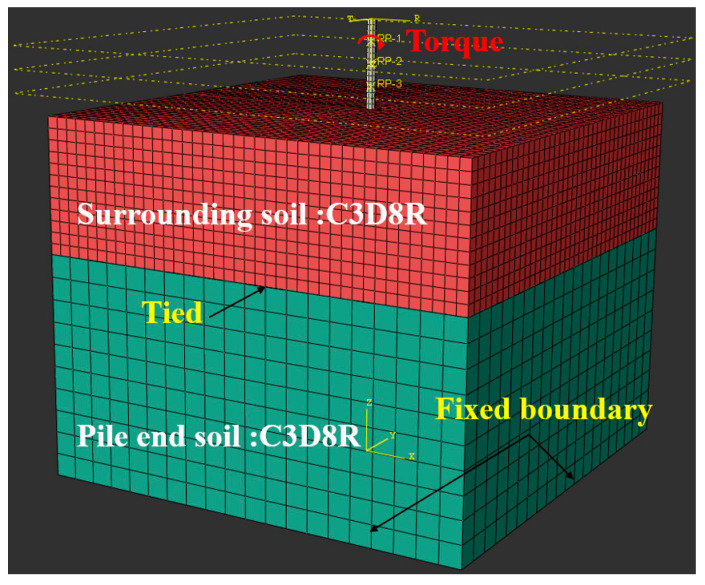
Mesh of the Finite element model.

To verify the accuracy of the proposed solution in simulating the simultaneously propagating upward and downward strain waves, the results calculated from the present model are compared with those computed from FEM. The finite element model is established and solved using Abaqus Explicit solver and C3D8R is utilized as the elements for both the soil and pile, the mesh of which is depicted in Figure 3. 

**Figure 4:**
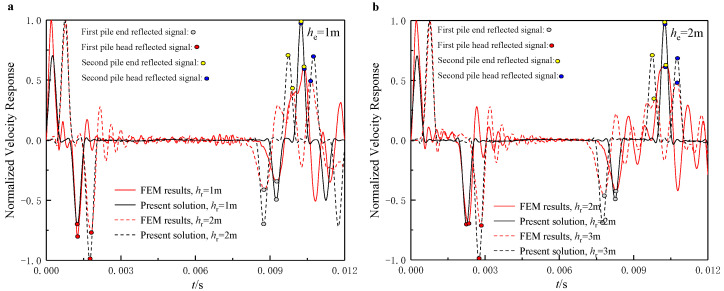
Comparisons of velocity response between present solution and FEM results: (**a**) $h_{e}=1\;m$; (**b**) $h_{e}=2\;m$.

As shown in Figure 4, the results derived from the present solution show good agreement with those calculated from the FEM, especially for the occurrence time of each reflection. However, there can be seen some deviations in the amplitudes of the reflected signal, mainly because of more significant strain wave energy dissipation in the 3D FEM model than in the present solution. In addition, by inputting the incident wave as close to the pile head as possible, the incident wave and the reflection of the upward wave at the pile head are more likely to be identified as one signal so that the difficulties of signal interpretation can be considerably reduced.

## 6. Parametric Studies

### 6.1. Layouts of the Input and Signal Receiving Locations

Based on the above analysis, a preliminary conclusion is drawn: by inputting the incident wave as close to the pile head as possible, the difficulty in signal identification and interpretation can be reduced. This section investigates the influence of the layouts at the input and signal receiving locations on the velocity response, aiming to find the optimal layouts for the TLSTs. In order to simulate the test for floating piles, the length of the fictitious soil pile is set as 5 m.

**Figure 5:**
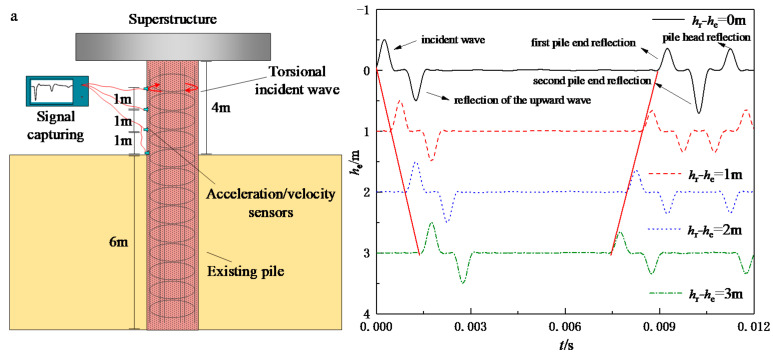

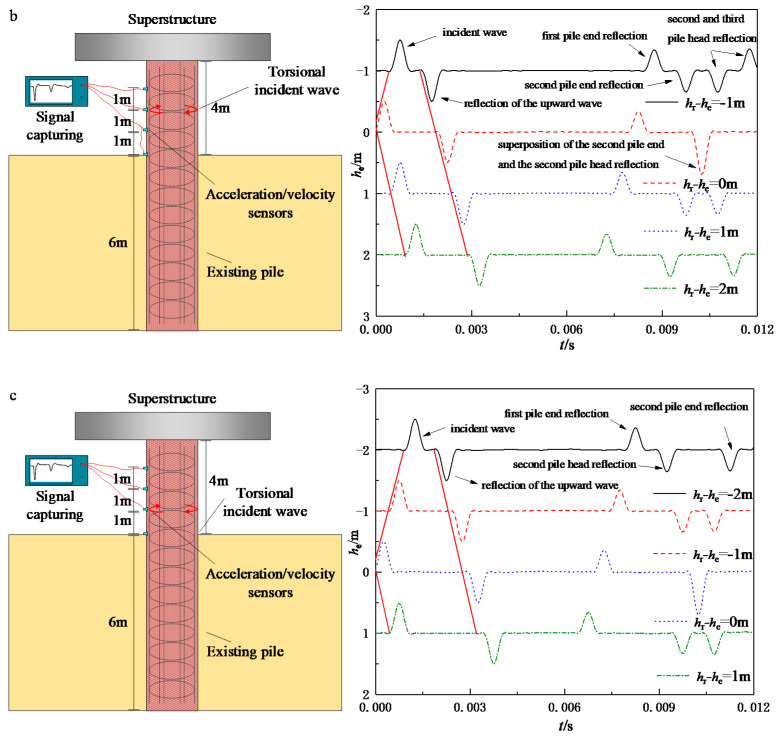
Influence of the layouts of the input and signal receiving locations on the TLSIT spectrums: (**a**) $h_{e}=1\;m$; (**b**) $h_{e}=2\;m$; (**c**) $h_{e}=3\;m$.

As shown in Figure 5, once the sensors are placed where the incident wave is applied, the number of the reflected signals is minimized, making the signal spectrum clearer. In addition, the time intervals between the incident wave and the reflection of the upward wave collected by the sensors installed above the input position of the incident wave would not vary with the changes in the input position. In contrast, the time intervals collected by the sensors installed below the input position would increase when the input position moves away from the pile head. For cases where inputting the incident wave close to the pile head is difficult, installing the sensors close to the pile head can be an alternative. However, the optimal layouts of the TLST are inputting the incident wave close to the pile head and installing the sensors close to the pile head as well.

### 6.2. Identification of Defects from the TLST Spectrums

Defect identification is one of the major tasks for the integrity examination of the existing pile foundations. Further, the neckings and concrete segregations are the two most commonly found defects in practice. This section investigates the identification ability of these two defects utilizing the TLSTs. 

**Figure 6:**
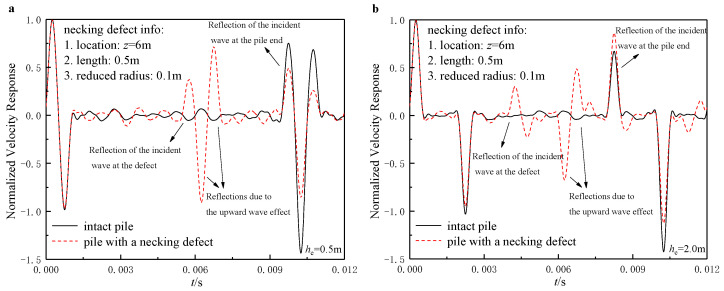
Identification of necking defects through TLSIT: (**a**) $h_{e}=0.5\;m$; (**b**) $h_{e}=2.0\;m$.

**Figure 7:**
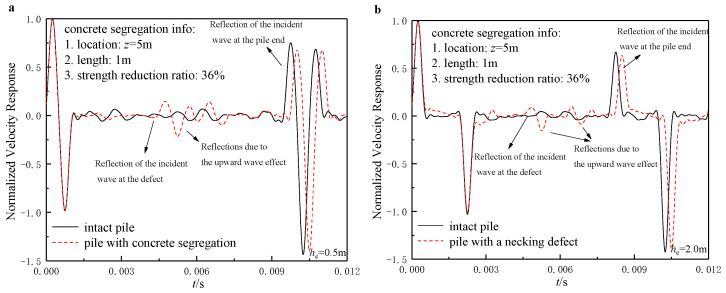
Identification of concrete segregation through TLSIT: (**a**) $h_{e}=0.5\;m$; (**b**) $h_{e}=2.0\;m$.

The results shown in Figure 6 and Figure 7 again justified the rationality of the optimal layouts of the TLSTs proposed in the above paragraphs. As shown in Figure 6a and Figure 7a, both the necking and concrete segregation defects can be clearly identified, as long as the incident wave is input close to the pile head and the sensors are installed close to the pile head as well. However, once the incident wave is input far from the pile head, identifying the reflected signals at the defect turns out to be extremely difficult because the reflected signals can no longer be identified as one signal but as several separate signals reflecting all the time, making the signal spectrum a mess. In addition, the concrete segregation defects would alter the pile body’s wave velocity so that each reflected signal’s occurrence time would vary, while the reflected signals caused by necking defects would not.

## 7. Conclusions

This paper establishes a rigorous mathematical model to simulate the strain wave propagation during the torsional low-strain test (TLST) for existing high-pile foundations. In the proposed model, the simultaneous propagation of the upward and downward strain waves inside the pile body is considered. The parametric analysis reveals the optimal layouts of the TLSTs for the existing high-pile foundation. The main conclusions can be drawn as follows:

1. By placing the sensors where the incident wave is applied, the number of reflected signals can be minimized to acquire a more precise signal spectrum.
2. The optimal layouts of the TLST are inputting the incident wave close to the pile head and installing the sensors close to the pile head as well. By doing this, the defects can be more easily identified from the signal spectrum.
3. The existence of concrete segregation defects would influence the occurrence time of each reflected signal, while the necking defects would not. Hence, this is a helpful tip for distinguishing the concrete segregation defects (decrease in strength of pile body material) from the necking defects.

## Figures and Tables

**Table 1:** Soil parameters utilized for model verification and parametric studies.

Density	Young’s Modulus	Poisson’s Ratio	Shear Modulus
1800 kg/m^3^	12 MPa	0.3	4.6 MPa

**Table 2:** Default pile parameters utilized for model verification and parametric studies.

Density	Young’s Modulus	Poisson’s Ratio	Shear Modulus	Length	Radius
2500 kg/m^3^	24 GPa	0.2	10 GPa	10 m	0.5 m

## Data Availability

The data presented in this study are available on request from the corresponding author.
